# Kinesiological Analysis of Stationary Running Performed in Aquatic and Dry Land Environments

**DOI:** 10.1515/hukin-2015-0103

**Published:** 2015-12-30

**Authors:** Cristine Lima Alberton, Stephanie Santana Pinto, Natália Amélia da Silva Azenha, Eduardo Lusa Cadore, Marcus Peikriszwili Tartaruga, Bruno Brasil, Luiz Fernando Martins Kruel

**Affiliations:** 1 Physical Education School, Federal University of Pelotas, Pelotas, RS, Brazil; 2 Physical Education School, Federal University of Rio Grande do Sul, Porto Alegre, RS, Brazil; 3 Physical Education School, Midwest State University of Parana, Guarapuava, PR, Brazil

**Keywords:** aquatic exercise, electromyography, immersion

## Abstract

The purpose of the present study was to analyze the electromyographic (EMG) signals of the rectus femoris (RF), vastus lateralis (VL), semitendinosus (ST) and short head of the biceps femoris (BF) during the performance of stationary running at different intensities in aquatic and dry land environments. The sample consisted of 12 female volunteers who performed the stationary running exercise in aquatic and dry land environments at a submaximal cadence (80 beats·min^−1^ controlled by a metronome) and at maximal velocity, with EMG signal measurements from the RF, VL, ST and BF muscles. The results showed a distinct pattern between environments for each muscle examined. For the submaximal cadence of 80 beats·min^−1^, there was a reduced magnitude of the EMG signal in the aquatic environment, except for the ST muscle, the pattern of which was similar in both environments. In contrast to the submaximal cadence, the pattern of the EMG signal from all of the muscles showed similar magnitudes for both environments and phases of movement at maximal velocity, except for the VL muscle. Therefore, the EMG signals from the RF, VL, ST and BF muscles of women during stationary running had different patterns of activation over the range of motion between aquatic and dry land environments for different intensities. Moreover, the neuromuscular responses of the lower limbs were optimized by an increase in intensity from submaximal cadence to maximal velocity.

## Introduction

Exercises performed in the aquatic environment have different physiological and biomechanical responses from those carried out on dry land due to the influence exerted by the physical properties of water on the human body. Water-based exercises performed in the vertical position are characterized by reduced cardiovascular responses (Kruel et al., 2013; Srámek et al., 2000), as well as lower impact upon the joints of the lower limbs (Alberton et al., 2013; Miyoshi et al., 2004) when compared to the same exercise performed on dry land. Therefore, they can be recommended to individuals of any age for rehabilitation or health promotion purposes. In addition, studies suggest that this type of exercise training may produce many beneficial effects on aerobic capacity, strength and flexibility when properly prescribed (Pinto et al., 2014; Takeshima et al., 2002).

Electromyographic activity (EMG) from the trunk, upper and lower limb muscles has been assessed during water-based exercises performed in the vertical position, such as shallow-water walking (Barela et al., 2006; Masumoto et al., 2008; Miyoshi et al., 2004), deep-water running (Kaneda et al., 2009; Masumoto et al., 2014), resistance (Bressel et al., 2011; Castillo-Lozano et al., 2014; Colado et al., 2013; Pöyhönen et al., 2001), and aerobic exercises (Alberton et al., 2011; Pinto et al., 2011).

Nevertheless, few studies have analyzed EMG activity over the range of motion during these different types of water-based exercises (i.e., temporal analysis). Barela et al. (2006) examined the EMG activity from the lower limbs and trunk muscles of adult men during shallow-water walking compared to dry-land walking at a self-selected comfortable pace, and these authors observed a different EMG pattern throughout the range of motion between these environments. Masumoto et al. (2014) described the pattern of EMG activity from the lower limb muscles of active runners over the range of motion while striding in deep water and land treadmill running at three submaximal intensities, and they observed a similar muscle activity pattern during running in both environments at matched heart rate conditions. In contrast, Pöyhönen et al. (2001) investigated the pattern of EMG activity from the thigh muscles during knee flexion-extension water-based resistance exercise during the range of motion of single and multiple repetitions at maximal velocity. EMG activation was purely concentric during the exercise performed in one single repetition; however, with multiple repetitions, there was a reduction in the EMG signals of agonist muscles in the middle of the range of motion, with early activation of antagonist muscles to decelerate the movement and change its direction in each phase of the movement (i.e., knee flexion or extension).

The aforementioned studies addressed specific aquatic exercises (i.e., shallow-water walking, deep-water running, water-based resistance exercise) and suggested the influence of the environment and intensity on the pattern of EMG signals from different lower limb muscles over the range of motion of exercise; however, no studies have undertaken kinesiological analysis of the lower limbs during water-based aerobic exercises. Thus, the neuromuscular activity of specific water-based aerobic exercises (e.g., stationary running) should be investigated and compared to those performed on dry land. The aim of this study was to analyze the EMG activity of the rectus femoris (RF), vastus lateralis (VL), semitendinosus (ST) and short head of the biceps femoris (BF) muscles, as well as the angular position of the hip over the range of motion during stationary running performed at different intensities in aquatic and dry land environments.

## Material and Methods

### Participants

Twelve young, physically active women volunteered to participate in the present study (age: 22.33 ± 0.57 years; body height: 1.63 ± 0.02 m; body mass: 59.11 ± 1.88 kg; fat mass: 26.9 ± 1.43%). The female gender was chosen due to the popularity of this type of exercise in this population. The participants were familiar with the aquatic environment and were free of any musculoskeletal, bone and joint diseases. All of the participants were required to sign an informed consent form which contained all of the information about the procedures and potential risks involved in participation in the research project.

### Procedures

This study was approved by the Ethics Committee of the Federal University of Rio Grande do Sul and was performed in accordance with the ethical standards established in the 1964 Declaration of Helsinki. An initial session was held to collect the participants’ physical characteristics. Body mass and height measurements were obtained using an analogue medical scale and a stadiometer (FILIZOLA; Sao Paulo, Brazil). Skinfolds were measured using a plicometer (LANGE; Cambridge, United Kingdom) to estimate corporal density, with the application of the protocol proposed by Jackson et al. (1980). The fat mass was estimated by means of the Siri (1993) equation.

After the initial session, all of the volunteers completed a training session to familiarize themselves with the exercise at the intensities and in the environments that would be later investigated during data collection. In addition, details regarding the range of motion and other information related to the exercises were explained. Stationary running was chosen for this study as it is widely used in typical water-based aerobic classes, and it has received attention in the recent literature (Alberton et al., 2011; Raffaelli et al., 2011). This exercise is characterized by a single stance and a swing phase. The subjects started the exercise in the standing position with their arms at the side of the body. The first phase corresponded to the right hip and knee flexion to 90° starting the swing phase, followed by the right hip and knee extension until the stance phase. This movement was repeated alternating the right and left limbs. The movement of the upper limbs was performed only to provide balance to the subjects. The exercise was performed at two intensities: a submaximal cadence of 80 beats·min^−1^ and maximal velocity. These intensities were chosen due to their use in previous studies (Alberton et al., 2011; Cassady and Nielsen, 1992).

The activity of the following four muscles on the body’s right side was obtained via surface EMG: RF, VL, ST, and BF. Hair was shaved at the electrode placement sites, and the skin in these areas was abraded and cleaned with alcohol to keep the inter-electrode resistance low (< 3 kΩ), which was measured before each session using a digital multimeter. Surface monopolar electrodes 15 mm in radii (model Mini Medi-Trace 100, Kendall Ag/AgCl; Tyco, USA) were placed in bipolar configuration over the belly muscle parallel to the orientation of the muscle fibers at 2 cm distal from the innervation zone (Rainoldi et al., 2004). The innervation zone of each muscle was determined with the aid of an electrostimulator (model EGF 4030, CARCI, Sao Paulo, Brazil). The inter-electrode distance was maintained at 30 mm (Beck et al., 2005). The reference electrode was positioned on the clavicle (Alberton et al., 2011).

For the water-based protocol, an insulation procedure was performed to avoid interference by artifacts due to the contact of the electrodes with water (Rainoldi et al., 2004), according to the method described in a previous study (Pinto et al., 2010). The insulation was applied using waterproof transparent adhesive tape (Tegaderm, 3M; St. Paul, Minnesota, USA). Silicone glue was placed at the exit point of the cables to prevent water from entering them. The cables and preamplifiers were fixed with adhesive tape. EMG signals were collected using a 14-bit electromyograph (model Miotool400, MIOTEC Biomedical Equipment; Porto Alegre, Brazil) with a common-mode rejection ratio of 110 dB and 2,000 Hz per channel, each with a 4-channel system. Data were transferred to an A/D converter before being uploaded onto a personal computer (Miograph software, MIOTEC Biomedical Equipment; Porto Alegre, Brazil). In addition, reflective markers were placed on the hip (greater trochanter) and on the knee (lateral femoral epicondyle) for filming. This procedure was performed to determine the angular position of the hip (AP_hip_) over the range of motion, based on the thigh segment movement relative to the vertical line. To align the EMG data with the AP_hip_, a light signal was triggered with the onset of EMG data collection. A video camera was positioned on the sagittal plane on the participants’ right side, at a distance of 3 m. The video was obtained through an underwater window in the side of the pool, using a video camera (model 50 Hz JVC GR-DVL9800, Mini DV Digital Camcorder; Japan) and a cold-cathode lamp to illuminate the reflective markers.

Maximum voluntary isometric contraction (MVC) tests were performed on dry land before the exercise protocol to estimate the maximal EMG amplitude for each muscle. These values were used for further normalization of the EMG signal. Duration of the test was set to 5 s for each muscle (DeLuca, 1997). Contraction angles were measured with a goniometer (CARCI; Sao Paulo, Brazil) and were adjusted so that they could be maintained during the MVC against manual resistance in both the flexion and extension directions, according to the protocol used in the study of Alberton et al. (2011). For the RF and ST muscles, the EMG signal was recorded with the participants in the supine position with 90° of right hip flexion. For the RF, the right knee was maintained at 90° of flexion, with the isometric contraction of the right hip flexors. For the ST muscle, the right knee was maintained at full extension (0°), with isometric contraction of the right hip extensors. Afterwards, the participants were maintained seated with 90° of hip and 70° of knee flexion to record the EMG signal from the VL (contraction of the right knee extensors) and BF (contraction of the right knee flexors) muscles.

The subjects performed the exercise protocol in dry land and aquatic environments, with a 2 h interval between environments. During this interval, the insulation procedure was performed. This order was chosen to preserve the electrodes’ insulation. Stationary running was performed in both environments at a submaximal cadence corresponding to 80 beats·min^−1^ and at maximal velocity. The submaximal cadence of 80 beats·min^−1^ was reproduced by a digital metronome (model MA-30, Korg; Tokyo, Japan). At the submaximal cadence, the participants performed the exercise for 4 min, with recording of the EMG signal and filming from the 3rd to the 4th min. At maximal velocity, the exercise was performed for 15 s, and the data were recorded throughout the execution of the movement. The intensities were performed with a 5 min interval between them. The land-based protocol was conducted in a room with temperature between 22 and 26°C. The water-based protocol was performed in a deep pool (2 m), and depth reducers were used to ensure that the participants were immersed between the xiphoid process (while standing) and the shoulders (during movement). The water temperature was maintained between 30 and 31°C.

### Data analysis

Software (Dvideow software, Laboratory of Biomechanics & Institute of Computing, UNICAMP; Campinas, Brazil) was used to reconstruct the reflective markers as two-dimensional coordinates. The reflective markers corresponding to the first 10 repetitions recorded from each exercise situation were digitalized either automatically or manually. These data were later filtered using a fifth-order lowpass Butterworth filter, with a cutoff frequency of 8 Hz, and they were then processed, generating graph files containing the AP_hip_ over time (Matlab software, version 5.3, MathWorks, Inc.; Natick, Massachusetts, USA). From these graphs, 5 of 10 repetitions, the peak AP_hip_ of which was within a range of ± 5° from the target angle (90°), were selected for each intensity and environment as this range was considered to be an adequate amplitude of motion. In the standing position, the reference value for complete extension of the hip and knee was 0°. Therefore, there was an average of five replicates for the presentation of the data. In addition, the starting and finishing time points for the subsequent EMG slices were obtained based on these five repetitions.

The digital EMG signal was filtered using a fifth-order band-pass Butterworth filter, with cutoff frequencies between 25 and 500 Hz (SAD32 software, Mechanical Measurements Laboratory, UFRGS; Porto Alegre, Brazil). The signal curves corresponding to the MVC (5 s) were sliced between 2 and 4 s to obtain the root mean square (RMS) value, which was used to normalize the EMG data (%MVC). The individual starting and finishing time points of each repetition, based on the AP_hip_, were used to create slices of the acquired EMG signal corresponding to each total repetition of the exercise. The values of the ranges for the RMS envelope were obtained from the calculation of 10% of the average time that the participants required to perform each cycle at different cadences. Thus, an interval of 150 ms was used for the cadence of 80 beats·min^−1^ for both environments as there was no difference in cycle duration between the environments (Table 1). For the maximal velocity, the intervals were 47 ms for dry land and 64 ms for aquatic environment, as there was a difference in cycle duration between environments (Table 1). These curves (i.e., RMS envelope) were normalized in time from 0 to 100%. Subsequently, the 5 selected repetitions were averaged to obtain the mean cycles for individual muscles for each participant in each situation. These curves were interpolated, and the same process was repeated to obtain the mean cycle for each muscle among the participants.

### Statistical analysis

The paired t-test was used to compare duration of the mean cycle between the aquatic and dry land environments for each intensity as the data presented a normal distribution. An alpha level of 0.05 was adopted, and SPSS software, version 20.0, was employed for this analysis. The mean values, corresponding to the EMG signal of each muscle over time (i.e., temporal analysis), as well as the mean AP_hip_, were determined.

## Results

**Figure 1:**
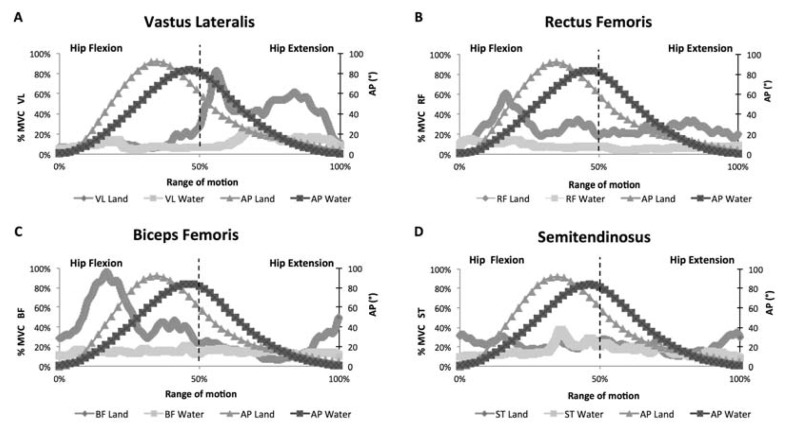
Pattern of the electromyographic signal of the vastus lateralis (A), rectus femoris (B), biceps femoris (C) and semitendinosus (D) muscles over the range of motion during stationary running performed at the submaximal cadence of 80 beats·min^−1^. Note: %MVC – percentage of maximum voluntary isometric contraction; AP – angular position of the hip; VL – vastus lateralis; RF – rectus femoris; BF – short head of biceps femoris; ST – semitendinosus.

At the submaximal cadence of 80 beats·min^−1^, the peak of the curve of AP_hip_, corresponding to approximately 90°, occurred earlier for both environments, i.e., before 50% of the cycle, particularly for the dry land environment, as shown in Figure 1. The RF, VL and BF muscles showed reduced EMG responses in the aquatic environment, while the ST muscle showed similar magnitudes between environments for the EMG responses (Figure 1). Regarding the phases, the VL muscle showed an increase in EMG activation in the second phase of the cycle (hip and knee extension) in both environments. The RF and BF muscles showed constant activation over the range of motion in the aquatic environment. In contrast, on dry land, the peak EMG activation of RF and BF muscles occurred in the first phase of the cycle (hip and knee flexion), when these muscles act as agonists. In addition, the ST muscle showed constant activation in the first and second phases for both environments.

**Figure 2:**
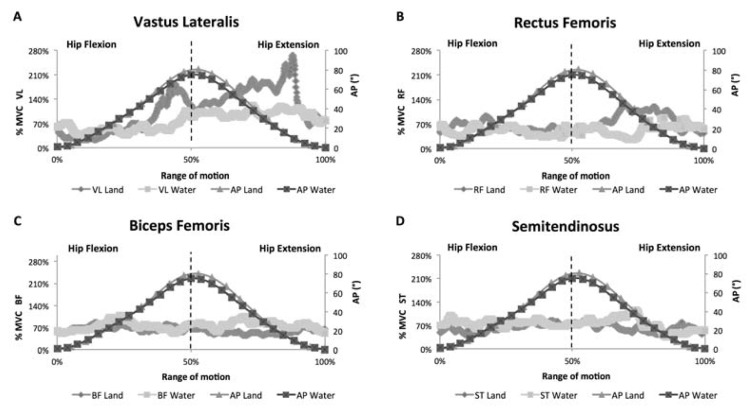
Pattern of the electromyographic signal of the vastus lateralis (A), rectus femoris (B), biceps femoris (C) and semitendinosus (D) muscles over the range of motion during stationary running performed at maximal velocity. Note: %MVC – percentage of maximum voluntary isometric contraction; AP – angular position of the hip; VL – vastus lateralis; RF – rectus femoris; BF – short head of biceps femoris; ST – semitendinosus.

At the maximal velocity, the peak of the curve of AP_hip_ occurred in the middle of the movement in both environments (Figure 2). By increasing the pace from submaximal to maximal, all of the examined muscles exhibited higher EMG activity. Moreover, in contrast to submaximal intensity, the magnitude of the difference between environments in the EMG signal from the RF and VL muscles was attenuated at maximal velocity, and no differences in the EMG pattern were observed between environments for the BF and ST muscles. In addition, both phases of the cycle presented similar EMG values in both environments, except for the VL muscle, the peak EMG activation of which occurred in the second phase of the cycle.

## Discussion

The main finding of the present study was the different pattern of EMG activation between aquatic and dry land environments for the RF, VL, BF and ST muscles over the range of motion of stationary running performed at different intensities. In addition, the peak of the curve of AP_hip_ occurred at different timepoints, according to the analyzed intensity and environment.

Stationary running, performed at submaximal intensity in both environments, presented the peak of AP_hip_ (hip flexion ≈ 90°) occurrence before 50% of the complete cycle. This result suggested a more pronounced stance phase in the end of the complete cycle, indicating the strategy adopted by participants to strike one foot on the ground before lifting the other foot, particularly on dry land, as, in the aquatic environment, the peak of AP_hip_ occurred too close to 50% of the cycle. The increase in intensity from submaximal to maximal altered the peak of AP_hip_ occurrence for the middle of the movement in both environments. These results indicated that, at maximal intensity, the stance phase was short and both the hip and knee flexion and extension phases of stationary running had similar duration. In addition, since duration of the cycle (swing and stance phases) was different between intensities (submaximal: 1.5 s; maximal: 0.5–0.6 s), the stance phase was shorter at maximal velocity than at submaximal cadence in an attempt to gain speed. These results were in accordance with those observed in the study conducted by Miyoshi et al. (2006), who assessed the stance phase of the gait during shallow-water walking, suggesting that the contact time was inversely proportional to velocity. Thus, the higher the intensity, the shorter the stance phase of the gait.

The comparison of the pattern of neuromuscular activity between environments over the range of motion of stationary running, performed at the submaximal cadence of 80 beats·min^−1^, showed lower EMG responses in water for the VL, RF and BF muscles. These data were consistent with previous studies (Alberton et al., 2011; Bressel et al., 2011; Kelly et al., 2000), which analyzed different water-based exercises performed with vertical displacement and which reported lower EMG responses for the aquatic environment than for the dry land environment. This lower EMG activity might be explained by the reduced hydrostatic weight (i.e., apparent weight) of individuals, accounting for a reduction ranging from 69 to 85% of total body weight when the body is immersed between the xiphoid process and the shoulders (Alberton et al., 2013; Harrison et al., 1992), which was the depth immersion used in the present study. This reduced apparent weight represents a lower resultant force to be supported and displaced in water in contrast to dry land, decreasing the energy expenditure during stationary exercises performed with vertical displacement of the limbs (Alberton et al., 2009). In contrast to these findings, aquatic exercises performed at submaximal intensities with horizontal displacement, such as shallow-water walking and deep-water running, activate some muscles more in the aquatic environment than on dry land, due to the higher drag force provided by the greater viscosity and density of water, compared to air (Alexander, 1977). In contrast, the ST muscle showed a similar pattern of EMG activation between the environments at both submaximal and maximal intensities. This finding might be explained by this muscle’s different functions in each environment. On dry land, the ST muscle is used only for the stance phase of the leg, while in the aquatic environment, it is also required during hip extension against buoyancy, as well as to overcome the drag force (Alberton et al., 2011).

When comparing the submaximal cadence with maximal velocity in the aquatic environment, the pattern of each muscle was changed, and the amplitude of the EMG signals from all of the examined muscles was increased. This result was observed in response to increased drag force (Fd), as shown by the general fluid equation: Fd = ½ ρA v2 Cd, where ρ is the fluid density, A is the projected frontal area, v is the velocity of movement, and Cd is the drag coefficient. Thus, an increase in the velocity of movement has great influence on water resistance, as velocity is squared and is directly proportional to resistance (Alexander, 1977), presenting an EMG signal that increases from 10–30% of MVC at 80 beats·min^−1^ to 50–100% of MVC at maximal velocity in different phases of the range of motion for the VL, RF, BF and ST muscles.

In addition, maximal velocity minimized the magnitude of differences in electrical activity from the lower limb muscles between environments during stationary running. Moreover, a different pattern of the EMG signal over the range of motion was observed, compared to the findings at submaximal cadence. In this situation, the VL muscle displayed a similar pattern of muscle activation to that in the submaximal cadence, with lower magnitude of reduction in the EMG signal in the aquatic environment during the second phase of movement (i.e., hip and knee extension). At both intensities, greater VL activation occurred in the second phase as this muscle is monoarticular and is an agonist of knee extension movement. In contrast, at maximal velocity, the RF, BF and ST muscles had similar magnitude and patterns of muscle activation between both phases and environments. This similar magnitude between environments confirmed the findings obtained by Alberton et al. (2011) and Kelly et al. (2000), who found no significant differences between environments for the EMG signals of the upper and lower limbs at high or maximal velocities. The authors suggested that this velocity seemed to be the point at which the buoyancy effect was suppressed by the drag force of water.

Moreover, the similar EMG magnitude between the phases observed for the RF, BF and ST muscles at maximal velocity in the aquatic environment might be attributed to both agonist and antagonist muscles, at high velocities of motion, being active in each phase of movement (e.g., flexion or extension) to overcome drag forces and to decelerate movement, respectively, as described in a previous study (Pöyhönen et al., 2001). This outcome occurs as, once the masses of water have been sufficiently accelerated, there is a decrease in the EMG activity of agonist muscles, and consequently, the antagonist muscles anticipate their activation to decelerate the movement and change the direction (Pöyhönen et al., 2001). This pattern suggests that, in water-based exercises performed at high velocities, the activation of the muscle groups involved in movement is first concentric (agonist muscles) and then eccentric (antagonist muscles) in each phase of motion (e.g., flexion or extension) (Pinto et al., 2011).

In summary, the EMG signals from the RF, BF, VL and ST muscles of women during stationary running had different patterns of activation over the range of motion between aquatic and dry land environments at the different intensities investigated in the present study. Moreover, the neuromuscular responses of the lower limbs were optimized by an increase in intensity (i.e., 80 beats·min^−1^ to maximal velocity). Thus, individuals who require lower EMG activity to protect the neuromuscular system might perform these water-based aerobic exercises, which are widely used in aquatic activities, at submaximal intensity. In contrast, healthy individuals who require an increase in their lower limb muscle activation might perform this exercise at maximal velocity in an aquatic environment. In this situation, similar magnitude of EMG activity of the evaluated lower limb muscles was found to that on dry land, with stretch-shortening type contractions, which are important in numerous daily activities (i.e., walking, running, jumping).

## Figures and Tables

**Table 1:** Comparison of duration of the mean cycle between aquatic and dry land environments for each intensity

Variable	Intensity	Dry land Environment		Aquatic Environment		Sig.

		Mean	SD	Mean	SD	

	80 beats·min^−1^	1.50	± 0.01	1.48	± 0.01	0.068
	Maximal velocity	0.47	± 0.01	0.64	± 0.01	0.003*

* p< 0.05
